# Alterations of White Matter Microstructure in Migraine Patients Vary in the Peri-ictal Phases

**DOI:** 10.1523/ENEURO.0300-24.2024

**Published:** 2025-01-10

**Authors:** Ana R. Fouto, Rita G. Nunes, Irene Guadilla, Amparo Ruiz-Tagle, Inês Esteves, Gina Caetano, Nuno A. Silva, Pedro Vilela, Raquel Gil-Gouveia, Patrícia Figueiredo

**Affiliations:** ^1^Institute for Systems and Robotics and Department of Bioengineering, Instituto Superior Técnico, Universidade de Lisboa, Lisbon 1049-001, Portugal; ^2^Universidad Autónoma de Madrid, Madrid 29049, Spain; ^3^Learning Health, Hospital da Luz, Lisbon 1500-650, Portugal; ^4^Imaging Department, Hospital da Luz, Lisbon 1500-650, Portugal; ^5^Neurology Department, Hospital da Luz, Lisbon 1500-650, Portugal; ^6^Center for Interdisciplinary Research in Health, Universidade Católica Portuguesa, Lisbon 1649-023, Portugal

**Keywords:** diffusion MRI (dMRI), diffusion tensor imaging (DTI), diffusional kurtosis imaging (DKI), microstructure, migraine, white matter

## Abstract

Alterations in white matter (WM) microstructure are commonly found in migraine patients. Here, we employ a longitudinal study of episodic migraine without aura using diffusion magnetic resonance imaging (dMRI) to investigate whether such WM microstructure alterations vary through the different phases of the pain cycle. Fourteen patients with episodic migraine without aura related with menstruation were scanned through four phases of their (spontaneous) migraine cycle (interictal, preictal, ictal, and postictal). Fifteen healthy controls were studied in the corresponding phases of the menstrual cycle. Multishell dMRI data were acquired and preprocessed to obtain maps of diffusion parameters reflecting WM microstructure. After a whole-brain analysis comparing patients with controls, a region-of-interest analysis was performed to determine whether the patients’ microstructural changes varied across the migraine cycle in specific WM tracts. Compared with controls, patients showed reduced axial diffusivity (AD) in several WM tracts across the whole brain in the interictal phase and increased fractional anisotropy (FA) in commissural fibers in the ictal phase. Interestingly, AD returned to baseline levels during peri-ictal phases in specific projection and association fibers. In contrast, FA values decreased in the ictal phase away from normal values in a few commissural and projection tracts. Widespread WM fiber tracts suffer structural variations across the migraine cycle, suggesting microstructural changes potentially associated with limbic and salience functional networks and highlighting the importance of the cycle phase in imaging studies of migraine.

## Significance Statement

Our study reveals dynamic changes in white matter (WM) microstructure across different phases of the migraine cycle, using advanced diffusion magnetic resonance imaging. By employing a case-control longitudinal design on patients with episodic menstrual migraine, we identified transient reductions in axial diffusivity and fractional anisotropy in specific WM tracts, which varied with migraine phases independently of hormonal influences. This is the first study to document such phase-dependent microstructural changes, highlighting the brain's ability to adapt rapidly to pain. Our findings provide significant insights into neuroplasticity, enhancing the understanding of migraine and potentially informing broader neurological research on the brain's response to pathological states.

## Introduction

Migraine is a neurological condition that causes cyclical episodes of mild to moderate headaches along with other neurological symptoms that can start before (preictal) and during (ictal) and persist after (postictal) the headache itself. The ictal phase, when the patient experiences the migraine attack, lasting for 4–72 h according to the third edition of the International Classification of Headache Disorders (ICHD-3; Olesen, 2018), can be disabling and making it difficult to complete daily tasks. Patients in the period between episodes, known as the interictal phases, are mostly asymptomatic. In episodic migraine, patients spend more time in the interictal than in the peri-ictal phases (Goadsby et al., 2017).

Neuroimaging research has contributed to a more comprehensive understanding of the complex brain changes in migraine (Rahimi et al., 2022; Schramm et al., 2023). Diffusion magnetic resonance imaging (dMRI) has been used to map water diffusion parameters reflecting the underlying microstructure. These include fractional anisotropy (FA), related with fiber integrity, and mean diffusivity (MD), related with tissue damage (Winston, 2012). More specifically, axial diffusivity (AD) can be used to assess axonal integrity by measuring diffusion along the primary axis, while radial diffusivity (RD) indicates myelin damage by measuring diffusion perpendicular to the primary axis (Winston, 2012).

Research on migraine using dMRI has primarily focused on white matter (WM) and the evaluation of patients during the interictal phase compared with controls, showing widespread abnormalities (Pierpaoli et al., 1996; Rahimi et al., 2022). Despite some contradictory findings, decreased AD has often been reported in multiple WM tracts (see review, Rahimi et al., 2022). However, changes in other diffusion parameters such as MD and FA have also been reported but with an inconsistent pattern across studies (Rahimi et al., 2022). Nevertheless, abnormalities have mostly been reported in the corpus callosum (Li et al., 2011; Yu et al., 2013b; Messina et al., 2015; Shibata et al., 2018; Szabó et al., 2018), thalamus (DaSilva, 2007; Yu et al., 2013a; Coppola et al., 2014), thalamic radiations (Yu et al., 2013b; Chong and Schwedt, 2015; Messina et al., 2015; Petrušić et al., 2018; Shibata et al., 2018; Szabó et al., 2018), superior and inferior longitudinal fasciculus (Yu et al., 2013b; Chong and Schwedt, 2015; Messina et al., 2015), and cingulate gyrus (Liu et al., 2013; Messina et al., 2015; Petrušić et al., 2018; Szabó et al., 2018).

Only two studies (Coppola et al., 2014; Marciszewski et al., 2019) have used a longitudinal design to examine changes during different phases of the migraine cycle, which focused on specific gray matter (GM) structures (i.e., brainstem nuclei and thalamus). One study (Marciszewski et al., 2019) found increased MD/AD during the interictal phase, which reverted to normal levels during the preictal phase and increased again after the attack, in brainstem nuclei (e.g., periaqueductal GM or cuneiform nucleus). The other study (Coppola et al., 2014) observed higher FA and slightly lower MD values in bilateral thalami in patients in the interictal phase compared with those in controls, which returned to normal in the ictal phase suggesting plastic peri-ictal modifications. Both studies suggest that there are fluctuations in local GM microstructure throughout the migraine cycle, but no research has been done on whether similar changes occur in WM brain pathways.

The aim of our study was to investigate alterations in WM microstructure of fiber bundles of migraine patients and during different stages of the migraine cycle. We focused on patients with episodic migraine without aura, with menstrual or menstrual-related migraine, in a longitudinal design, compared with a group of healthy controls (HCs) matched for the menstrual phase.

## Materials and Methods

### Study population

A group of 14 female patients with episodic migraine without aura with menstrual or menstrual-related migraine, which were diagnosed according to the ICHD-3, were recruited by a neurologist during the medical appointment at the headache outpatient clinic at the Hospital da Luz. Besides the diagnostic criteria, other inclusion criteria consisted of the following: (1) female; (2) adults 18–55 years of age; (3) at least 9 years of education and Portuguese native speaking; (4) otherwise healthy (i.e., not currently diagnosed with any disease that significantly impedes an active and productive life nor with a life expectancy below 5 years); and (5) not currently receiving treatment with psychoactive drugs (including anxiolytics, antidepressants, antiepileptics, and any migraine prophylactics). Exclusion criteria were as follows: (1) presence of aura; (2) record of neurologic disease other than migraine; (3) breastfeeding, pregnancy, or after menopause; and (4) contraindications for MRI. Moreover, the use of contraception was not mandatory. The following clinical variables were recorded for all patients: disease duration (in years), attack frequency (days per month), and pain intensity (visual analog scale, VAS). For the control group, we recruited 15 age-balanced healthy native Portuguese female volunteers through advertisement in the general population. They met the same criteria for inclusion and exclusion, with the only difference being that they did not experience any type of migraine or recurring headaches, defined as fewer than one headache episode per month in the last year, and these headaches do not meet the criteria for migraine according to the ICHD-3.

The study was approved by the ethics committee of the Hospital da Luz, and informed written consent was obtained from all participants according to the Declaration of Helsinki seventh revision.

### Study design

**Figure 1.**
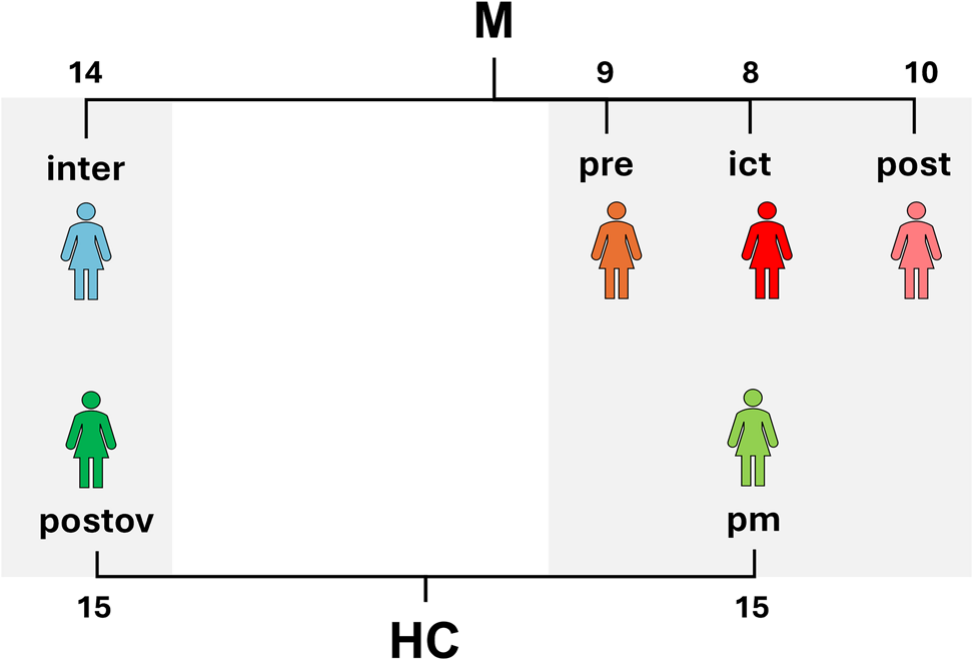
Illustrative schematics of the study design. In our study, migraine patients (M) were assessed in four MRI sessions based on the migraine cycle phase: interictal (M-inter), pre-attack (M-pre), during the attack (M-ict), and postattack (M-post). In addition, HCs were scanned in corresponding periods of their menstrual cycle: perimenstrual phase (HC-pm) and postovulation phase (HC-postov). The total number of subjects per session is shown.

Figure 1 illustrates the study design. All migraine patients (M) were evaluated longitudinally in four sessions corresponding to the four phases of the migraine cycle, namely: (1) interictal (M-inter); (2) preictal (M-pre); (3) ictal (M-ict); and (4) postictal (M-post; Peng and May, 2020). The experimental sessions order was counterbalanced to ensure that an equal number of the first sessions occurred during the peri-ictal (preictal, ictal, and postictal) and the interictal phases. The M-pre and M-post sessions were prescheduled in accordance with the patients’ menstrual cycle, while the M-ict sessions were planned during a patients’ self-reported migraine attack. The verification of each session was conducted in the following manner: (1) M-inter, if patients indicated that they had a complete absence of pain for a minimum of 48 consecutive hours before the experimental session and confirmation of the absence of a migraine attack was obtained 72 h after the session; (2) M-pre, up to 72 h before the start of a spontaneous migraine attack (Peng and May, 2020), confirmed with the volunteer afterward; (3) M-ict, was defined as occurrence of a migraine attack satisfying the ICHD-3 criteria; and (4) M-post, up to 48 h after the end of a spontaneous migraine attack (Peng and May, 2020), which was confirmed with the volunteer afterward. Regarding the alignment of the four sessions with phases of the menstrual cycle, we considered the following: M-inter, the period after ovulation, lasting up to 5 d; M-pre, the period up to 5 d before menstruation; M-ict, typically occurring between Days 0 and 1; and M-post, the period up to 4 d after the onset of menstruation.

The HCs were evaluated in two sessions corresponding to the phases of the menstrual cycle matching the interictal and the peri-ictal phases of the patients, respectively (Dubol et al., 2021): (1) postovulation phase, up to 5 d after ovulation (HC-postov), and (2) perimenstrual phase, from 5 d before to 5 d after menstruation (HC-pm). The experimental sessions order was counterbalanced to ensure that an equal number of the first sessions occurred during HC-pm and HC-postov. Furthermore, in both samples, the number of participants using hormonal contraception was evenly distributed. In addition, both groups of participants were evaluated for anxiety using State–Trait Anxiety Inventory (STAI; Spielberger, 1970) and for depression using Zung Self-Rating Depression Scale (ZSDS; Zung et al., 1965) questionnaires before scanning during M-inter and HC-postov sessions. Finally, age and literacy levels between patients and controls were compared using the Mann–Whitney *U* test, as the data were not normally distributed on JASP (www.jasp-stats.org/, version 0.17.3.0). Additionally, we compared the anxiety and depression score between groups using a Mann–Whitney *U* test.

### Data acquisition

In each study session, participants were scanned on a 3 T Siemens Vida system using a 64-channel RF-receiver head coil. MRI data were acquired over a 3 year period (June 2019 to December 2022), and all scanning sessions were performed in the evening (7:00–9:00 P.M.). The MRI protocol included various types of images; here, we report only the dMRI study, including the required anatomical T_1_-weighted scan and fluid-attenuated inversion recovery (FLAIR) for WM lesion detection.

#### dMRI data

We obtained multishell dMRI data using 2D echoplanar imaging and the following imaging parameters: 66 axial slices with an in-plane Generalized Autocalibrating Partially Parallel Acquisitions (GRAPPA) factor of 2, simultaneous multislice (SMS) factor of 3, and 2 mm isotropic resolution. The repetition time (TR)/echo time (TE) parameters were 6,800/89 ms. The diffusion-weighted images were acquired with *b* values of 400, 1,000, and 2,000 s/mm^2^ along 32, 32, and 60 unique gradient directions, respectively. Additionally, eight nondiffusion-weighted (*b*0) images were also collected as well as three *b*0 images with opposite phase encoding (posterior–anterior). The total acquisition time was 15:47 min.

#### Anatomical scans

We also acquired a T_1_-weighted structural image using a 3D magnetization-prepared rapid gradient echo sequence with the following parameters: TR, 2,300 ms; TE, 2.98 ms; inversion time (TI), 900 ms; total acquisition time, 5:12 min; flip angle, 9°; and field-of-view (FOV), 256 × 240 × 128 mm^3^, with 1 mm isotropic resolution. Finally, we acquired a FLAIR sequence with the following parameters: TR, 5,000 ms; TE, 386 ms; TI, 1,800 ms; total acquisition time, 5:57 min; GRAPPA, 2; flip angle mode, T_2_ var; and FOV, 240 × 240 × 151 mm^3^, with 0.9 mm isotropic resolution. Both anatomical scans were evaluated by an experienced neurologist.

### Data analysis

All dMRI data were preprocessed following the DESIGNER (Ades-Aron et al., 2018) pipeline. Firstly, denoising was performed using “dwidenoise” (Veraart et al., 2016), followed by Gibbs ringing correction using “mrdegibbs” (Kellner et al., 2016) and Rician bias correction (Koay and Basser, 2006). These steps were carried out using MRTrix tools (www.mrtrix.org/, version 3.0.3; Tournier et al., 2019). Subsequently, geometric distortions due to *B*_0_ inhomogeneities and eddy currents, as well as head motion, were corrected using FSL's tools “topup” and “eddy” (www.fsl.fmrib.ox.ac.uk/fsl/docs/#/, version 6.0.5; Andersson et al., 2003; Andersson and Sotiropoulos, 2015). Finally, bias field correction was performed using “dwibiascorrect” with *-ants* option from MRTrix.

After preprocessing, we used two different diffusion signal representations to estimate maps of multiple diffusion parameters: the commonly used DTI and an extension of this representation also including the diffusional kurtosis imaging (DKI; Jensen et al., 2005) tensor. For conventional DTI, we extracted the b1000 shell to estimate FA, MD, AD, and RD with *dtifit* from FSL. For DKI, we used both b1000 and b2000 shells, and besides the DTI parameters, mean kurtosis (MK), axial kurtosis (AK), and radial kurtosis (RK) were also estimated, using DESIGNER. All maps were nonlinearly aligned to the standard FA template (1 mm isotropic resolution FMRIB58_FA) using FNIRT from FSL. Each diffusion parameter map was then skeletonized through restriction to the main WM fibers and thresholded at 0.3, using the tract-based spatial statistics (TBSS) (Smith et al., 2006) tool in FSL.

#### Comparison between patients and controls

To investigate voxelwise differences in the diffusion parameters, we performed permutation tests on the skeletonized diffusion parameter maps, using the “randomise” tool from FSL (Nichols and Holmes, 2002). This statistical testing approach is theoretically supported (Nichols and Holmes, 2002) and widely used in the literature for its robustness in TBSS studies (Smith et al., 2006; Winkler et al., 2014). We utilized 5,000 permutations along with threshold-free cluster enhancement correction to account for multiple comparisons (Smith and Nichols, 2009), and the significance level was set at *p* < 0.05. We employed a two-group unpaired difference design, including age as a covariate, to perform the following comparisons between patients and controls in matching sessions: (1) [M-inter] versus [HC-postov]; (2) [M-pre] versus [HC-pm]; (3) [M-ict] versus [HC-pm]; and (4) [M-post] versus [HC-pm]. As a control analysis, we employed a single-group paired difference design to compare the two sessions of the controls: [HC-pm] versus [HC-postov].

#### Variations across the migraine cycle

To investigate differences between the four phases of the migraine cycle in patients, we performed a region-of-interest (ROI) analysis across 28 ROIs defined by combining each pair of corresponding right and left WM tracts from the ICBM-DTI-81 WM atlas (Hua et al., 2008). First, we extracted the median of each diffusion parameter within each ROI, for each participant and session. Then, for each diffusion parameter in each ROI, we obtained the parameter change for each patient and session relative to controls, by calculating the difference between each patient/session and the average across controls in the respective session, i.e., (1) ΔM−inter=[M−inter]−[HC−postov]; (2) ΔM−pre=[M−pre]−[HC−pm]; (3) ΔM−ict=[M−ict]–[HC−pm]; and (4) ΔM−post=[M−post]– [HC−pm]. Finally, we fitted a linear mixed-effect model to the parameter change values using the R program (www.r-project.org/, version 1.3.1056), using cycle as a fixed effect with four levels (M-inter, M-pre, M-ictal, and M-post) and the patients as a random effect. This statistical approach is well-suited for handling missing data, in this way providing potentially greater statistical power by considering all available data points from each patient. Post hoc analysis was performed when significant main effects were found, using Benjamini–Hochberg correction for multiple comparisons across ROIs (*p* < 0.05). Only the DKI parameters were retained for the ROI analysis because they demonstrated greater sensitivity than DTI to identify changes between groups (see Results).

#### Correlation with clinical variables

We assessed whether the previously calculated relative parameter differences during each phase of the migraine cycle for each diffusion parameter were correlated with the clinical variables’ disease duration, attack frequency, and pain intensity by computing the Spearman correlation using the JASP software.

## Results

### Study population and MRI data

Both age and literacy levels were equivalent between the 14 female patients (age, 36 ± 9 years; literacy, 17 ± 2 years) and the 15 female controls (age, 30 ± 7 years; literacy, 17 ± 1 years). The *p* values (age, *p* = 0.11; literacy, *p* = 0.62) indicate no significant differences between the patients and controls in these parameters. Table 1 summarizes the information of the distribution of hormonal contraception use, anxiety scores, and depression scores. None of the participants had any daily medication other than contraception. Six migraine patients and five controls were taking combined oral contraceptives; three migraine patients had the copper intrauterine devices, while all the remaining participants used barrier methods. Migraine's average disease duration was 19 ± 10 years, having an average frequency of two attacks per month, with an average intensity of 7 ± 1 on the VAS (0–10). There were no significant differences between groups in terms of anxiety or depression scores. Both participant groups were classified as having “moderate anxiety” based on the STAI-T scores and reported “no depression” according to the ZSDS.

**Table 1.** Distribution of contraception methods and median (interquartile range) scores for anxiety (STAI-T) and depression (ZSDS) in migraine (M) and healthy control (HC) groups with corresponding *p* values

	M	HC	*p* value
Contraception			
None	5	12	-
IUD	3	0	-
Pill	6	3	-
Anxiety			
STAI-T score	38 (7)	40 (7)	0.41
Depression			
ZSDS score	38 (7)	36 (10)	0.46

IUD, intrauterine device.

A total of 30 datasets were collected from 15 HC, while 41 datasets were collected from 14 patients. All the recruited controls successfully completed both sessions, whereas only five patients were able to complete all four MRI sessions. The total number of concluded sessions among the patient cohort was as follows: 9 preictal, 9 ictal, 10 postictal, and 14 interictal. One ictal session was discarded because the number of volumes was incomplete. The duration of MRI data acquisition (defined as the number of days between the first to the last sessions) ranged between 63 and 689 d for patients (four sessions) and between 14 and 326 d for controls (two sessions).

Regarding the evaluation of anatomical scans, no relevant lesions were identified by the neurologist upon inspection.

### Comparison between patients and controls

**Figure 2.**
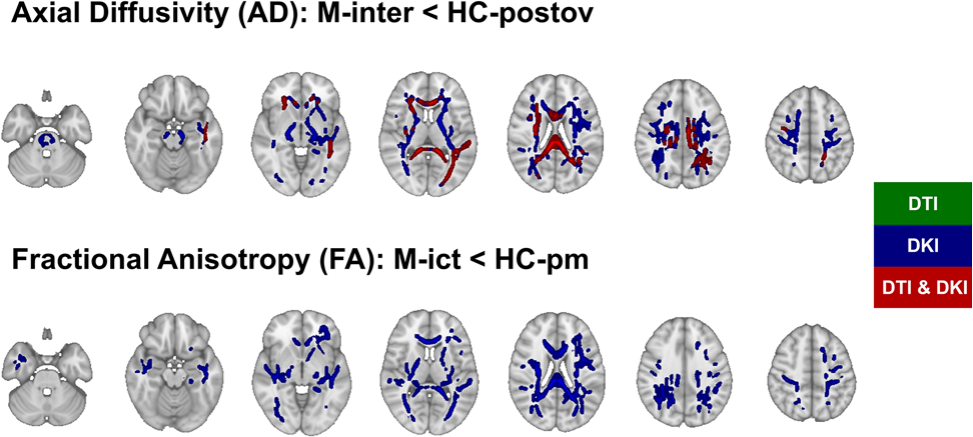
Spatial distribution of the significant differences found in the voxelwise comparisons between patients and controls: AD for [M-inter] < [HC-postov] (top) and FA for [M-ict] < [HC-pm] (bottom). The *p* value maps are displayed for DTI (green) and DKI (blue), as well as their overlap (red). Interictally, patients showed lower AD compared with HCs (in postovulation) across multiple WM regions, more with DKI. Ictally, patients showed lower FA compared with HCs (in perimenstrual phases) across multiple WM regions, only with DKI.

**Figure 3.**
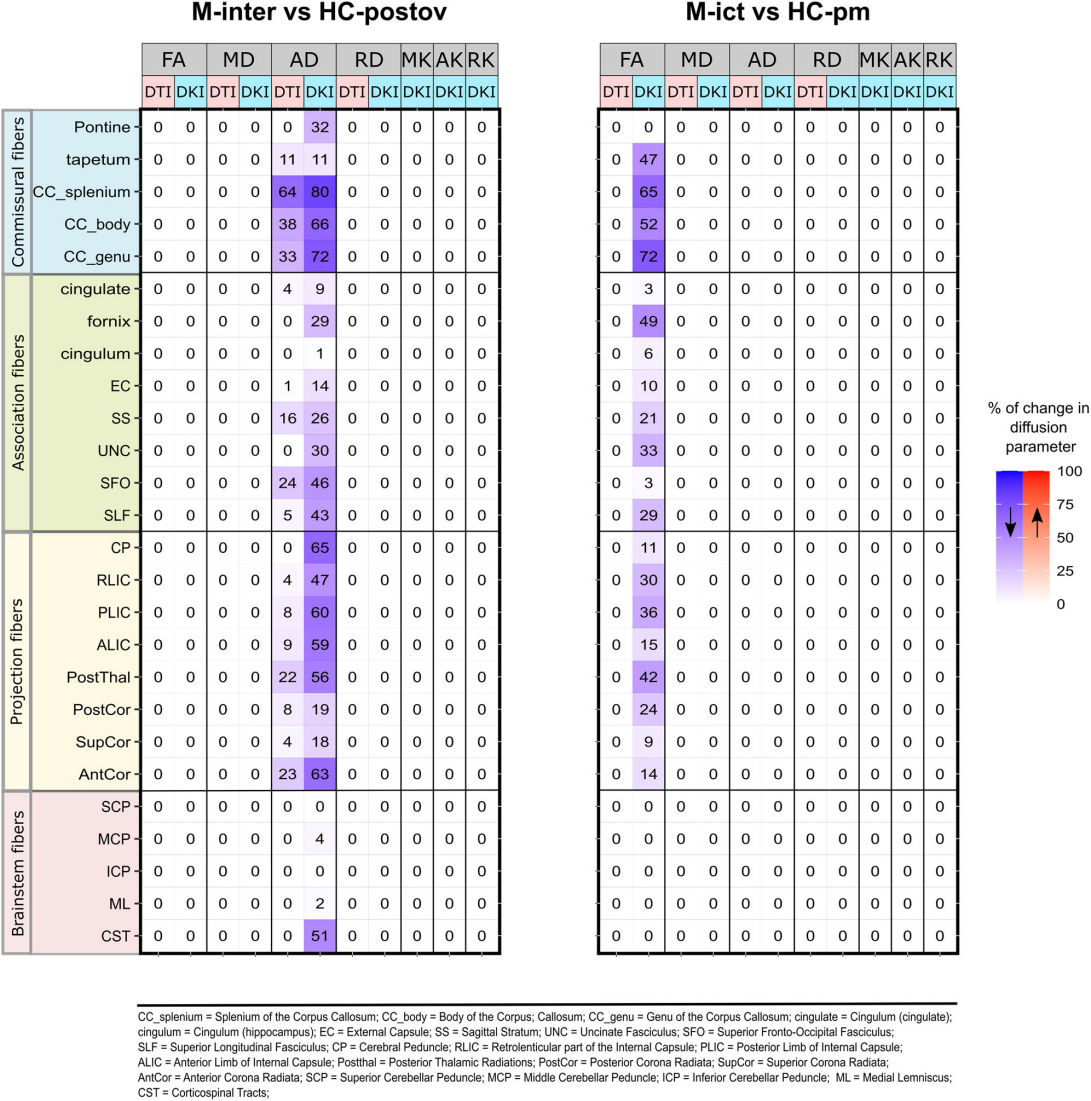
The percentage of voxels exhibiting significant changes in each ROI of the WM skeleton, for the two comparisons showing significant differences, [M-inter] versus [HC-postov] (left) and [M-ict] versus [HC-pm] (right), for each diffusion parameter (FA, MD, AD, RD, MK, AK, and RK), and both signal representations, DTI and DKI. Blue/red colors are used to represent decreased/increased values in comparison with controls.

The results of the voxelwise pairwise comparisons between patients and controls in each of the phases of the migraine cycle are summarized in Table 2, for both DTI and DKI methods. Only two comparisons showed significant effects in some parameters: M-inter versus HC-postov and M-ict versus HC-pm. DKI was more sensitive than DTI, yielding more significant differences: decreased AD, for M-inter versus HC-postov, and decreased FA, for M-ict versus HC-pm. Using DTI, only the decreased AD for M-inter versus HC-postov was detected. Figure 2 shows the spatial distribution of significant differences on the skeletonized diffusion parameter maps, and Figure 3 presents the percentage of voxels displaying significant differences within each WM tract of the ICBM-DTI-81 WM atlas, for each of the comparisons yielding significant differences. We quantify the extent of significant differences in terms of the percentage of voxels exhibiting differences. We interpret larger percentages as indicating a greater ability to detect subtle changes. Decreased AD estimated in M-inter was the most robust finding, obtained both using DTI and DKI. The effects of the covariate age in the comparisons between patients and controls are presented in Extended Data, for both DTI and DKI methods, including their spatial distribution (Extended Data Fig. 1-1) as well as the percentage of voxels displaying significant effects in the whole WM skeleton (Extended Data Table 1-1) and within each WM tract of the ICBM-DTI-81 WM atlas (Extended Data Fig. 2-1). Negative effects of age were found for MD, AD, and RD, especially for MD in the comparison M-ict versus HC-pm when using DTI. Positive effects of age were for found for MK and AK, especially for AK in the comparison M-inter versus HC-postov when using DKI. Because of the generally higher sensitivity of DKI compared with DTI to detected changes (of FA and AD) in patients relative to controls, only the DKI representation was used for the subsequent ROI analysis across the migraine cycle.

**Table 2.** Summary of the percentage of voxels exhibiting significant differences in the comparisons between groups (M and HC) and sessions (inter, postov, pre, pm, ict, post) for each diffusion parameter (FA, MD, AD, RD, MK, AK, and RK) and both diffusion models (DTI and DKI)

Comparisons	M-inter vs HC-postov		M-pre vs HC-pm		M-ict vs HC-pm		M-post vs HC-pm	
	DTI	DKI	DTI	DKI	DTI	DKI	DTI	DKI
FA	-	-	-	-	-	↓ (28%)	-	-
MD	-	-	-	-	-	-	-	-
AD	↓ (11%)	↓ (36%)	-	-	-	-	-	-
RD	-	-	-	-	-	-	-	-
MK	-	-	-	-	-	-	-	-
AK	-	-	-	-	-	-	-	-
RK	-	-	-	-	-	-	-	-

The results are corrected for age effects. Groups and sessions: M-inter, migraine interictal; M-pre, migraine preictal; M-ictal, migraine ictal; M-post, migraine postictal; HC-postov, healthy control postovulation; HC-pm, healthy control perimenstrual. Diffusion models: DTI, diffusion tensor imaging; DKI, diffusional kurtosis imaging. Metrics: FA, fractional anisotropy; MD, mean diffusivity; AD, axial diffusivity; RD, radial diffusivity; MK, mean kurtosis; AK, axial kurtosis; RK, radial kurtosis. Symbols: ↓, decrease; ↑, increase; -, no significant change.

Extended Data Figure 3-1 displays the spatial distribution of results from the voxelwise comparison between HCs. The comparison between the perimenstrual and postovulation sessions of the controls are displayed in Extended Data Figure 4-1. Significant decreases in MD, AD, and RD were found in the perimenstrual phase compared with postovulation phase, in several WM tracts across the whole brain, with also increased values in FA. These differences highlight the importance of controlling for the menstrual phase when analyzing the effects of the migraine phase, as done in the following section.

### Variations across the migraine cycle

**Figure 4.**
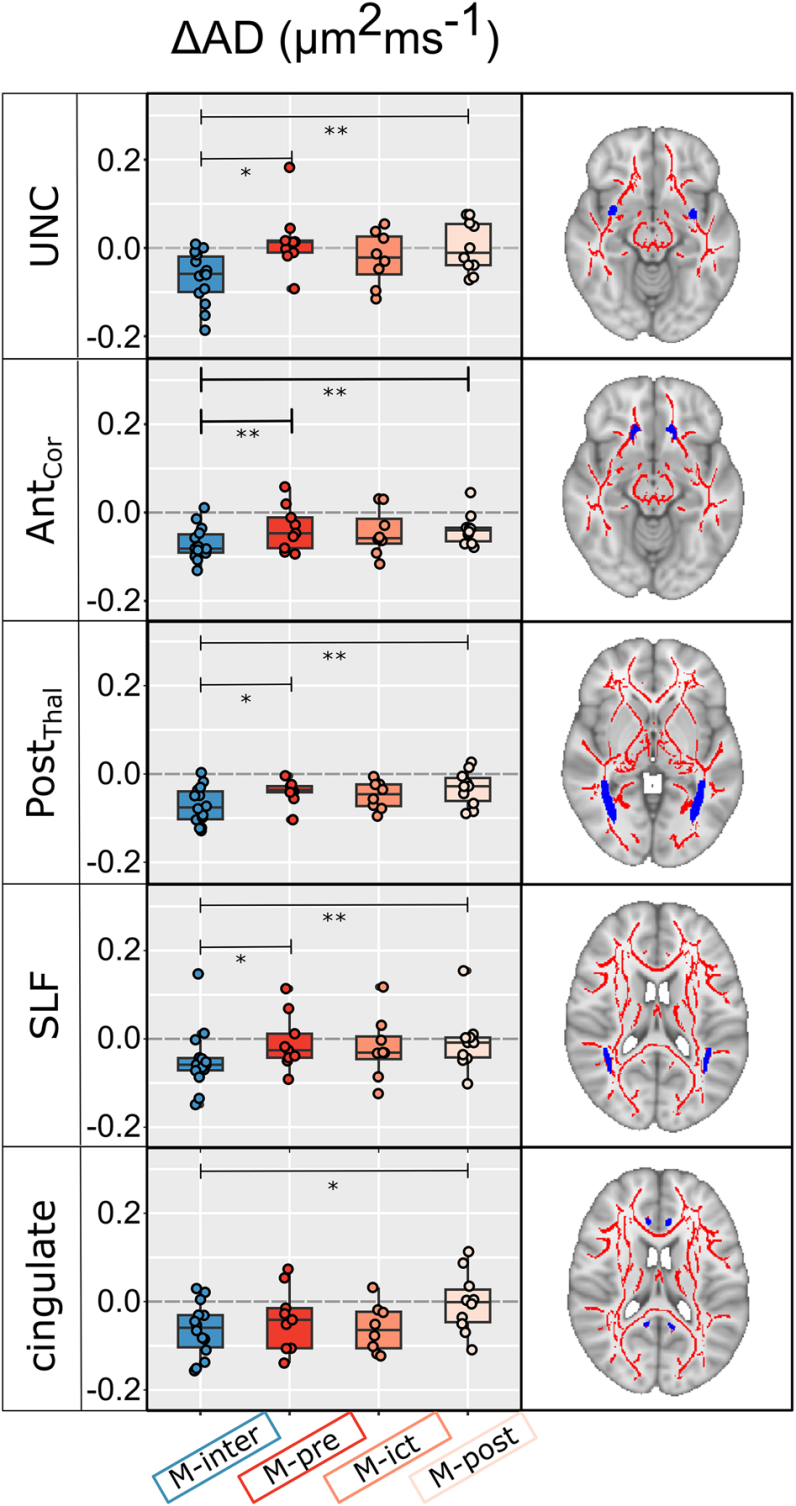
ROI analysis results are shown for AD exhibiting significant effects in multiple WM tracts across the migraine cycle estimated using the DKI signal representation. In the UNC, AD showed decreased values in the interictal phase, which tended to normalize during the peri-ictal phases. In addition, a reduction in AD was observed in the M-inter phase also in Ant_Cor_, Post_thal_, SLF, and cingulate, which also tended to normalize during the peri-ictal phases. Significant post hoc differences are shown and adjusted for multiple comparisons as indicated by *p < 0.05; **p < 0.01; ***p < 0.001.

**Figure 5.**
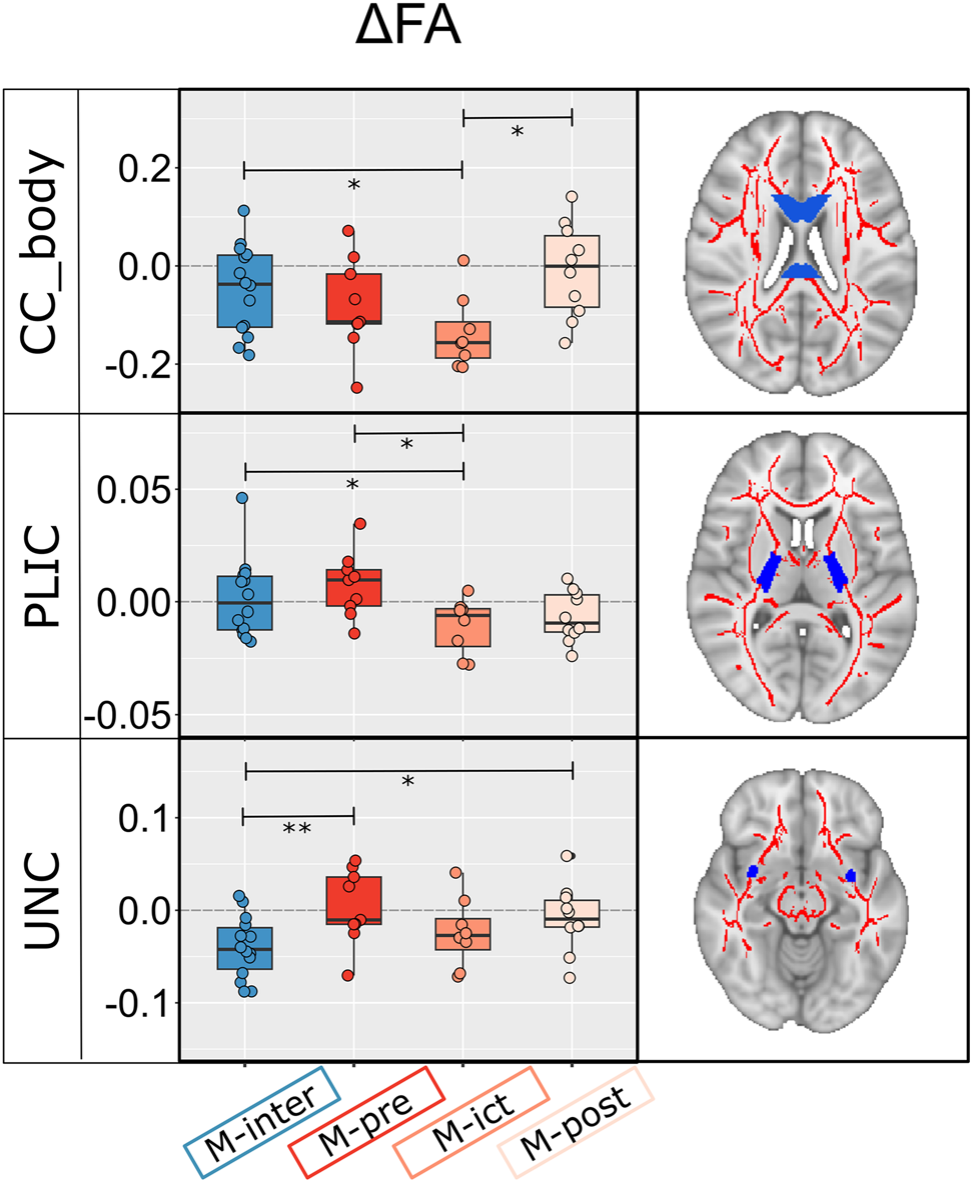
ROI analysis results are shown for FA exhibiting significant effects in multiple WM tracts across the migraine cycle estimated using the DKI signal representation. In the UNC, FA showed decreased values in the interictal phase, which tended to normalize during the peri-ictal phases. In addition, FA in both the PLIC and CC_body showed normal values in the M-interictal phase, but these were significantly reduced specifically in the M-ictal phase. Significant post hoc differences are shown and adjusted for multiple comparisons as indicated by *p < 0.05; **p < 0.01; ***p < 0.001.

The ROI analysis of the diffusion parameters, where changes in patients were identified relative to controls (estimated using DKI), revealed significant main effects of the migraine cycle phase in five ROIs for AD and three ROIs for FA, as presented in Figures 4 and 5, respectively. In a few WM tracts, decreased AD values during the headache-free period (M-inter) tended to return to normal values during the peri-ictal phases when they became comparable with those observed for controls: uncinate fasciculus (UNC), anterior corona radiata (Ant_Cor_), posterior thalamic radiations (Post_thal_), superior longitudinal fasciculus (SLF), and cingulate (cingulum cingulate gyrus). For FA, both the posterior limb of the internal capsule (PLIC) and the body of the corpus callosum (CC_body) showed normal values in the interictal phase, but these were significantly reduced specifically in the ictal phase. Significant variations of FA during peri-ictal phases were also found in the UNC.

### Correlation with clinical variables

The clinical parameters were correlated with the values of FA and AD that were extracted from each WM fiber tract yielding significant effects of the migraine cycle. No significant correlations were observed among the variables. Only the following variables showed some correlation trends (that did not survive correction for multiple comparisons): negative correlation between the frequency of attacks and AD (M-inter) in Ant_Cor_; disease duration and FA (M-pre) in Post_thal_; and disease duration and AD (M-ict and M-pre) in CC_body (Extended Data Fig. 5-1).

## Discussion

For the first time, we document variations in WM microstructure across different phases of the migraine cycle in episodic migraine patients without aura, specifically focusing on menstrual or menstrual-related migraine. Our study, which includes comparisons with HCs during matched menstrual cycle phases, reveals significant microstructural changes in both interictal and ictal phases. We found reductions in AD, MD, and FA in specific WM fibers, aligning with previous findings (Rahimi et al., 2022). Notably, we also found that AD and FA changes were phase-dependent, suggesting WM microstructure fluctuations throughout the migraine cycle. Our study is distinguished by its use of the advanced method DKI, which enabled us to uncover differences that could not be detected by conventional DTI, representing a significant advance in the literature with only one study reporting DKI results in the periaqueductal GM (Ito et al., 2016).

### Microstructural changes in patients versus controls

Our study found significant changes in brain structure in patients compared with controls during the respective menstrual phases. There was a decrease in certain diffusion parameters (FA and AD) without a decrease in MD and RD during both ictal and interictal phases. These results align with a previous study on migraine patients (Yu et al., 2013a). Additionally, using DKI, we found no differences in MK, AK, and RK. These findings are inconsistent with another study on migraine patients using DKI analysis (Ades-Aron et al., 2019).

Although both DTI and DKI detected reductions in AD in the interictal phase, DKI was more sensitive in the detection of AD changes (36 vs 11% of voxels). Moreover, only DKI was able to detect changes in FA in the ictal phase. Indeed, DKI may offer enhanced sensitivity for detecting subtle and complex microstructural changes, which could be particularly relevant in conditions such as migraine. Overall, changes in diffusion parameters (FA and AD) suggest alterations in axonal integrity, possibly due to short-term membrane permeability changes and shifting axonal geometries (Benedetti et al., 2011; Shua et al., 2011). The lack of significant differences in radial parameters (RD and RK) suggests that the observed changes may not be primarily linked to permanent myelin degeneration or plastic changes in axonal diameter, density, or fiber organization.

The differences in FA and AD results between M-ict and HC-pm identified by DKI may stem from several factors. DKI employs a more sophisticated model than traditional DTI, capturing non-Gaussian diffusion and offering enhanced sensitivity to subtle microstructural changes. Moreover, DKI generally utilizes a higher number of diffusion gradient directions, improving data resolution and accuracy. These methodological improvements could account for the distinct diffusion metrics observed between the migraine and control groups, even with similar parameters. During the interictal phase, microstructural changes were found in several brain regions of migraine patients compared with controls. More than 50% of voxels in projection fibers like the posterior thalamic tract, anterior corona radiata, internal capsule, and cerebral peduncle showed alterations, as well as the corpus callosum and brainstem. In the ictal phase, over 50% of voxels in the corpus callosum showed differences. The corpus callosum consistently displayed impairment in both phases, possibly due to its role in pain control regulation and information transmission (Li et al., 2011). However, previous studies have not consistently reported these findings, and the extent of changes varied across different tracts (Rahimi et al., 2022). Nonetheless, our results emphasize that employing DKI or more advanced diffusion models beyond DTI is advantageous for detecting additional or complementary WM changes that might not be captured by conventional methods.

Some effects of age were found in the analysis including patients and controls. In particular, with age we found reductions in MD (particular RD) and increases in kurtosis (particularly AK). Although the limited age range of the participants and the small sample size of our study preclude a robust analysis of the effects of age, it was important to account for age in our comparison between patients and controls because their age distributions were not exactly matched (the difference was not significant, probably as a consequence of the small sample size). Indeed, microstructural changes with age have been reported (Davis et al., 2009; Inano et al., 2011). Our findings partially align with previously reported results. Specifically, we also observed that RD is more sensitive to the integrity of myelin, indicating that alterations in WM associated with aging are predominantly caused by the degeneration of myelin rather than axons (Davis et al., 2009).

### Microstructural variations across the migraine cycle

Our study found transient and rapid changes in the microstructure of certain WM fiber tracts in migraine patients throughout the migraine cycle. Importantly, these changes are distinct from natural hormonal variations observed in controls during the menstrual cycle. Specifically, reductions in certain parameters of the UNC were observed during the interictal phase, which normalized during the peri-ictal phases. Similar patterns of AD were observed in other tracts, such as thalamic projection tracts and association fibers.

These findings underscore the importance of considering the timing of imaging in relation to the migraine cycle when interpreting diffusion parameters, as previous studies that assessed patients at varying and sometimes poorly defined phases may have resulted in inconsistent findings. Such inconsistencies could stem from studies focusing on different stages, such as ictal or interictal, or from ambiguous phase definitions, leading to contradictory results.

The variation in diffusion characteristics over the migraine cycle suggests short-term structural changes affecting water mobility and the microenvironment around WM tracts. At the onset of a migraine episode, a temporary normalization or repair of the microstructure may occur. These changes may reflect either preexisting or adaptive neuronal changes, indicating a predisposition to migraine attacks where the brain becomes easily hyperexcitable (Karsan and Goadsby, 2023). In this context, our study is reminiscent of the findings by Schulte et al. (2020), who observed hypothalamic activation using fMRI in the 48 h preceding migraine episodes, hence highlighting the presence of short-term functional changes alongside the short-term microstructural changes we observed. Similar cyclical patterns have also been observed in other fMRI studies, particularly in brainstem and thalamic structures (Marciszewski et al., 2019). Additionally, other aspects of brain activity and behavior, such as auditory evoked potentials and spinal trigeminal nuclei responses to pain stimulation, also exhibit cyclical behavior with normalization preceding migraine attacks (Ambrosini et al., 2003; Stankewitz et al., 2011).

The involvement of certain fiber bundles in different phases of the migraine cycle suggests a connection to cognitive and behavioral symptoms associated with migraines. For example, the UNC, linking the orbitofrontal cortex to the anterior temporal lobes is involved in cognitive functions such as language, memory, and social–emotional processing (Von Der Heide et al., 2013). This implies that examining episodic memory and social–emotional processes in relation to decision-making influenced by emotions or behaviors is relevant (Olson et al., 2015). Similarly, SLF and cingulate gyrus also show fluctuations throughout the migraine cycle and are connected to the anterior cingulate cortex (ACC). The ACC is part of the salience network and plays a role in processing the importance of stimuli when making decisions, especially in the context of pain (Rolls, 2019). Furthermore, differences in functional connectivity within the ACC have been observed between episodic and chronic migraine patients, suggesting its involvement in migraine chronification (Gomez-Pilar et al., 2022). Longitudinal studies have also shown cyclical changes in functional connectivity in the limbic and salience networks, with a “brain reset” mechanism occurring during migraine attacks (Stankewitz and Schulz, 2022). These findings suggest that the altered functionality of these networks may be influenced by changes in WM fiber pathways (Schulte et al., 2020).

Overall, our findings suggest that migraine is a disorder with not only short-term functional changes but also short-term structural changes. Specifically, they support the hypothesis that a predisposition to migraine episodes is associated with changes in pain processing regions of the ACC and trigeminal pathways (Goadsby et al., 2017). Understanding these functional and structural underpinnings may facilitate the development of targeted therapies for migraine pathophysiology, such as interventions focused on emotion regulation, sensory modulation, and cognitive training, which may be particularly effective in managing migraine attacks and associated symptoms.

### Methodological considerations

To ensure the reliability of our study, we made several methodological considerations. We focused exclusively on otherwise healthy young women with episodic migraine without aura with menstrual or menstrual-related migraine offering a distinct advantage over previous studies that included different migraine or without appropriate control for relevant factors that influence WM microstructure (e.g., as biological sex and hormonal state, age, or medication; DaSilva, 2007). We also matched the controls for sex, age, literacy, and menstrual cycle phase to minimize potential confounding factors related to hormonal changes in the brain (Dubol et al., 2021).

The choice of studying episodic patients with menstrual or menstrual-related migraine allowed not only a good distinction of the ictal and interictal periods but also a reasonable prediction of attacks, which was critical for capturing spontaneously occurring attacks, one of the challenges of studying the migraine cycle (May, 2009). Many cross-sectional studies overlook the longitudinal aspect of migraine, but our study design involved scanning patients throughout the four phases of the pain cycle. We believe this approach would offer a more comprehensive understanding of microstructural changes occurring over time. The control group consisted of women whose menstrual cycle phase was monitored, allowing us to isolate the effects of the menstrual cycle on brain diffusion and analyze the impact of hormonal fluctuations on brain function.

In terms of imaging technique, we utilized DKI in addition to the more conventional DTI. DKI offers advantages over DTI because it models non-Gaussian diffusion, which allows it to capture more complex microstructural features potentially leading to more detailed results compared with DTI. While multishell data could potentially improve DTI results by providing additional diffusion information, we used the b1000 shell to align with standard analysis practices and ensure comparability with established methods. Therefore, the superior performance of DKI in our study is attributed to its advanced modeling of diffusion, not to limitations of the b1000 data itself. We employed suitable statistical analysis methods to enhance reliability, specifically TBSS, to analyze the whole-brain maps acquired for each diffusion parameter. While both DTI and DKI detected a reduction in AD, DKI was more effective in capturing other differences. In fact, our results highlight the increased sensitivity of DKI in identifying migraine-related microstructural alterations that DTI cannot, supporting its potential as an imaging biomarker for migraine.

Indeed, our study employed two analysis methods: TBSS analysis to evaluate whole-brain WM changes and ROI analysis to evaluate specific WM tracts. This dual-method strategy aimed to enhance the robustness and sensitivity of the study, capturing both global and focal differences in WM integrity. Therefore, by combining both methods, it provides detailed insights into localized changes and maximizes the sensitivity to detect subtle alterations in the brain regions involved in migraine pathophysiology.

### Limitations

The study has several limitations that need to be acknowledged. The small sample size makes it difficult to draw definitive conclusions, and future studies should confirm the findings. This is difficult to overcome given the longitudinal study design capturing spontaneous attacks which presents challenges in larger sample sizes. However, the results are consistent with previous research in terms of transversal changes between patients and controls.

Additionally, the fact that not all patients completed the four sessions of the study limited the power of the longitudinal study design to assess fluctuations in diffusion parameters across the pain cycle. The study was impacted by the COVID pandemic, which affected collaboration and access to the scanner. Despite these limitations, some patients were able to complete the study, demonstrating the feasibility of conducting long-term research in a clinical setting.

Another limitation is the lack of quantification of hormonal fluctuations, which could be a confounding factor in menstrual-related migraine. Future studies should consider incorporating hormonal tracking to better understand the interplay between hormones and migraine-related brain changes. Although efforts were made to match hormonal cycles between patients and controls, the wide ranges of days corresponding to different phases of the migraine cycle may have obscured finer hormonal effects. On the other hand, while the study effectively controlled for the migraine phase, other variables such as sex and age were not as rigorously controlled. This study included only women and did not match age distributions precisely, but it is important to emphasize the effective control of the migraine phase and hormonal state.

## Conclusion

We have, for the first time, demonstrated longitudinal variations in the diffusion parameters of WM fibers across the migraine cycle. Our results reveal significant structural changes in widespread WM tracts, which appear to be associated with functional networks. These findings underscore the importance of considering the phase of the migraine cycle in imaging studies.

While further research is needed to elucidate the biological mechanisms underlying these cyclic alterations, our study highlights the potential for future investigations to target specific phases of the migraine cycle and improve the understanding of its impact on WM microstructure.

## Data Availability

The data that support the findings of this study are available from the corresponding author, upon reasonable request.

## Synthesis

Reviewing Editor: Jennifer Dulin, Texas A&M University Decisions are customarily a result of the Reviewing Editor and the peer reviewers coming together and discussing their recommendations until a consensus is reached. When revisions are invited, a fact-based synthesis statement explaining their decision and outlining what is needed to prepare a revision will be listed below. The following reviewer(s) agreed to reveal their identity: Ãlvaro Planchuelo-GÃ³mez.

This study reports dynamic microstructural white matter changes in human subjects with menstrual migraine that occur across different phases of the pain cycle. The findings of this study highlight the brain's ability to adapt rapidly to pain states and enhances the understanding of migraine-associated structural changes. Both reviewers agreed that this is an original study describing novel data that are of broad interest to the neuroscience community. Reviewers noted the use of Diffusion Kurtosis Imaging as a strength of the study, as it is infrequent in the migraine literature. However, some major weaknesses limit enthusiasm for the manuscript. These include sample size, differences in age distribution of the migraine and control groups, and other questions about statistics. Please see point-by-point comments by reviewers, below.

Reviewer #1:

This study, titled "Alterations of white matter microstructure in migraine patients vary in the peri-ictal phases", presents dMRI longitudinal data to study WM microstructure alterations in patients with episodic menstrual migraine. They observed migraine phase-dependent microstructural changes in some specific white matter tracts, highlighting the brain's ability to adapt rapidly to pain. However, before drawing this conclusion in publication, the authors need to evaluate the experimental designs and data analysis more thoroughly. Below are my major points.

Major points

1) All the patients and controls are females in this study. Why only female subjects are included, what is the rationale? [Editor's note: Please disregard this comment - I recognize that the nature of the studied topic requires that all subjects must be females.]

2) For DTI and DKI results, DKI results are significantly better than DTI results. Why is that? What are the possible underlying mechanisms? The DTI calculated in this study was based on b1000 shell data, however, DTI can also be calculated based on multi-shell data. If multi-shell DTI was calculated, what would be the results?

3) Figures 5 and 6 compared DKI results across different migraine cycles in multiple WM tracts. However, only 5 patients were able to complete all four MRI sessions. To track the longitudinal data, Figures 5 and 6 may only need to include these 5 patients, all statistics should be performed for each patient.

4) Figures 2 and 3 showed the spatial distribution of the significant differences found in two migraine phases. To confirm the robustness of these results, bootstrapping should be done by randomly picking some data from the whole dataset and calculating the robustness of the results from each picking data.

Reviewer #2:

The aim of this work was to assess the white matter structural changes of female patients with menstruation-related episodic migraine without aura across the migraine cycle and taking into account the hormonal cycle. To reach their objective, the authors conducted a whole-brain analysis using diffusion tensor imaging (DTI) and diffusion kurtosis imaging (DKI). On the one hand, the authors compared patients and healthy controls using the tract-based spatial statistics technique, controlling the results for the migraine phase and the menstrual cycle. On the other hand, comparisons between the migraine patients were carried out considering the diverse phases of the migraine cycle. The main strength of this study is the novelty of the results in association with the study of the phases of the migraine cycle using diffusion MRI techniques, incorporating the use of DKI. The main weaknesses of this study are the sample size and, in the current version, the lack of details for certain parts and discrepancies between the text, figures and tables (especially Table 1). To sum up, I consider that this manuscript can be an interesting contribution to the diffusion MRI migraine literature, although some corrections are necessary to highlight the novelty of the study and some major concerns I have.

Major concerns

Introduction

1. I think that paragraphs 2 and 3 can be summarised without losing relevant information, as there is some redundancy. For instance, there are two consecutive sentences showing that dMRI and DTI are useful to detect microstructural abnormalities, and they could be perfectly merged. Something similar happens with the relationship between water diffusion parameters and microstructure. The text occupied by the definition of the DTI parameters and the potential clinical relevance of each one is long, considering the information or the relationship with glial cells, which can be considered as an overinterpretation of the results from previous studies. The "mathematical" definition or relationship with water diffusion of the parameters (not the clinical interpretation) can be moved to the Methods. To summarise the interictal WM changes previously reported, the authors can refer to global changes in the mentioned regions, and perhaps move the clinical interpretation to the paragraph describing the previous studies assessing the migraine cycle. Furthermore, it is strange for me that the most consistent finding of the literature is reduced AD in patients with migraine, and not reduced FA, which should be the most reported result. Certainly, the opposite FA trend has been reported, but also the opposite AD trend (Kattem Husøy et al., 2019; DOI: 10.1186/s10194-019-1028-6).

Materials and Methods

2. What questionnaire was employed to evaluate anxiety and depression? Had any participant a score associated with anxiety or depression? Further information about the results of the questionnaire can be provided in the Results section, including the distribution of specific scores or the qualitative classification (e.g., subjects with and without depression according to the questionnaire).

3. How long did it take to acquire the MRI data for patients (the four or the available sessions) and controls (the two sessions)? To add this information, the authors should consider the distribution of time between first and last acquisition, and the longest value between both timepoints, considering all subjects.

4. I have some doubts about the permutation tests described in the Comparison between patients and controls. Just reading the manuscript, it seems that the statistical analysis of the comparisons between migraine patients and controls was the same as the comparison between the two sessions of the controls. The first set of comparisons must follow a two-group difference design (like a two-sample unpaired t-test), while the comparisons between the two sessions of the controls must follow a single-group paired difference design (paired t-test). If the designs were right, this information would just be added to the manuscript. Otherwise, apart from including this information in the manuscript, the comparison or set of comparisons with wrong design must be repeated and the results corrected.

Results

5. The age distribution of patients and controls was not close. If there was no significant p-value comparing the age of both groups, it could be related to the small sample size rather than actual lack of differences. Moreover, considering the comparisons between controls and subsets of migraine patients, it would be convenient to check the age distribution for each migraine subgroup. In any case, I suggest adding a new set of comparisons between patients and controls, correcting the results for age. If results were similar to the original after adding age as covariate of no interest, then the reported differences can be associated with migraine or migraine phases. If the statistically significant differences disappeared, the reported changes could be associated with aging effects rather than migraine or migraine phases. Something similar may happen for effects associated with anxiety and/or depression.

6. Lines 210-212. DKI may present higher sensitivity than DTI, but for me it is not "clearly greater". There are comparisons with voxels showing statistically significant differences only for DTI (MD in Figure 2 is a good example), and in Figure S1, FA differences were only found with DTI. Thus, the authors must use a more moderate language to show the advantages of DKI. It is right to justify the exclusive use of DKI for the assessment across the migraine cycle, but the interpretation between brackets must be included in the Discussion, not in the Results (a reference to the Discussion can be made for higher details).

Discussion

7. I would partially rewrite the first paragraph to highlight the novelties or strengths of this study. There are three main aspects that should be additionally remarked in my opinion: 1) menstrual or menstrual related-migraine (read comment 25 for further details); 2) use of DKI to find differences unidentified with DTI (as clearly shown in Figure 3); 3) the structural changes depending on the phases of the migraine cycle. To my knowledge, only one journal paper has reported DKI findings in migraine, but in the periaqueductal grey matter (Ito et al. 2016, doi: 10.1007/s00234-015-1603-8), and the associated reference cited by the authors was a conference paper, considering that most headache experts, most likely, have no access to ISMRM papers. Certainly, the last sentence referred to the third point I listed, but I would add that, for the first time, this study showed structural white matter changes through the phases of the migraine cycle.

8. The second subsection of the Discussion is related to functional MRI results. In this context, this study reminds me the paper by Schulte et al. (2020; doi: 10.1002/ana.25697). In this work, migraine patients were daily scanned using fMRI for 30 days or more, and the authors found activation of the hypothalamus in the 48 hours before the migraine episode. Therefore, considering the results from this study and the one by Schulte et al. (and the other references), I would emphasise that migraine is a disorder with not only short-term functional changes, but also short-term structural changes. In the last paragraph of the subsection, if the authors referred to structural and functional changes, mention both explicitly (or what the authors wanted to describe).

Conclusions

9. I find right to remark that longitudinal changes across the migraine cycle were found for the first time. However, I would use a more direct style and make reference to more specific results. The authors can use the end of the abstract to add information to the conclusion, and separate future studies from the impact of this paper. In other words, use separate sentences to highlight the potential of the study and the line of future studies.

Figures and Tables

10. Table 1. The results of the comparison between M-inter and HC-postov are inconsistent with the text and Figure 2. For DTI, taking Figure 2 as the reference, in the patients, reduced MD and AD were found with DTI, but the Table showed reduced AD and RD, and the text only mentioned decreased AD. Regarding DKI, the text and Figure 2 described reduced FA, MD and AD, and increased MK and AK in the patients, but the Table contained information about reduced AD and RD. The authors must make the necessary corrections wherever is pertinent or clarify the discrepancies.

Minor concerns

Abstract

11. For the last sentence, instead of "We report", I suggest using a direct style just showing the "consequence" of the results obtained by the authors, for instance, "Widespread structural WM tracts suffer structural variations across the migraine cycle, suggesting..." Regarding the keywords, instead of "episodic migraine" and "white matter tracts", I recommend "migraine" (it is more frequent than episodic migraine, and usually "migraine" involves "episodic migraine") and "white matter". For DTI and DKI, could the authors check some examples from the literature and use the most frequent exact terms? For example, "diffusion tensor imaging" is likely more employed than "diffusion-tensor imaging (DTI)".

Introduction

12. First paragraph. In my opinion, the last sentence can be removed (the one citing Stovner et al.), as it presents a completely different idea with respect to the previous text. This manuscript is not focused on migraine epidemiology, so this information is not crucial. Moreover, I suggest rewriting the sentences of lines 34-35 ("The ictal phase..." and "In between episodes..."). For the first sentence, I think that a more formal version would be more convenient, replacing "which is the actual migraine attack" by "when the patient experiences the migraine attack" (or something equivalent), and "and make it difficult" by ", making difficult". As this manuscript is focused on migraine stages, I would also define the duration of the ictal phase, 4-72 hours according to the third edition of the International Classification of Headache Disorders. For the other sentence, I think it would be better to replace "In between episodes" by "The period between episodes". As this journal is not specific of headache disorders, I suggest adding a brief description of episodic migraine.

Materials and Methods

13. Use of contraception is not an actual inclusion or exclusion criterion, if it is not mandatory. It is a relevant information that can be moved after the inclusion and exclusion criteria. I guess inclusion criterion c) is related to Portuguese native speaking, considering that the volunteers were native Portuguese subjects.

14. Can the authors provide the approval code of the ethics committee?

15. Checking the age distribution of patients and controls, I would state that controls were balanced for age, not age-matched (same for Discussion sections using the same term).

16. For the DKI dataset with b1000 and b2000 shells, were the DTI parameters obtained with DESIGNER?

17. Lines 123-126. The information about distribution of hormonal contraception and participants with and without anxiety and depression can be included in the Results section. The information about the questionnaire employed to evaluate anxiety and depression must be left in the Materials and Methods section.

18. Regarding the MRI acquisition, if T2-weighted or FLAIR images were obtained, include the corresponding sequence. Furthermore, if any of the two modalities was used for a radiological examination, this information should be added, especially in relation to white matter hyperintensities (WMHs). If there was no inspection of the WMHs, a frequent radiological finding in migraine, this should be included as a limitation of the study, as they may have a potential effect on the results, although their role in migraine is unclear. Regarding the diffusion-weighted images, the authors should clarify if 124 unique gradient directions (no repeated directions) and axial slices were employed. For all sequences, the authors should include the information about the flip angle. Also, define the abbreviations for TR, TE and TI when used for first time (I am not sure whether these abbreviations are obvious for the readers of this journal). Finally, the authors should add the approximate total acquisition time of the described MRI sequences, and the period when the images were acquired, especially considering that one of the limitations is related to the COVID-19 period.

19. How was the final sample size reached? Certainly, it is difficult to recruit many patients considering the restrictive conditions, but was any previous estimation conducted? Did the COVID-19 lockdown period influence the sample size?

20. The authors should add the version of the software tools, i.e., MRtrix, FSL and R. For the dMRI preprocessing, add the specific tools (e.g., dwidenoise for denoising).

21. For the comparisons between patients and controls, the tool for permutation tests is "randomise". This should be mentioned, and the right reference must be added (Nichols and Holmes, 2002; doi: 10.1002/hbm.1058).

22. Regarding the comparisons across the migraine cycle, not only the combined left and right regions, but also tracts without left and right divisions, such as the parts of the corpus callosum according to the ICBM-DTI-81 White-Matter Atlas, were employed. Which FDR correction procedure was employed? The authors should clarify these points in the manuscript.

Results

23. Lines 184-185. Which comparisons are associated with the written p-values? For me, it is unclear. Age and literacy? Why would these variables be compared? Were the age and literacy compared between patients and controls?

24. Assuming that the Results from Table 1 are partially right, regarding the comparison between M-ict and HC-pm, could the FA and AD results identified by "DKI" be related to distinct methods to fit the diffusion tensor? Or is this related to the use of more data and a higher number of diffusion gradient directions? A brief paragraph about these differences, despite using the same parameters, should be included in the Discussion.

Discussion

25. In the first paragraph, I would remark that the patients suffered from episodic migraine patients without aura, with menstrual or menstrual-related migraine, as it was written in the last paragraph of the Introduction. The menstrual migraine was precisely one of the novelties of this study, and it should be highlighted, in my opinion. Diffusion white matter changes in patients with episodic migraine without aura, without considering the menstrual migraine, had been already reported, as the results in Figures 2-4, and the novelty would be unclear if the menstrual part were not cited.

26. Regarding the first subsection of the Discussion, in the first paragraph, after the reference to the conference paper by Ades-Aron et al., to conclude the paragraph, I would add the idea that the use of DKI or more advanced models than DTI is helpful to identify additional or complementary diffusion white matter changes.

27. Second subsection. I find interesting the idea described in the last sentence of the first paragraph, but I think it could be rewritten in a more direct way to highlight the proper sentence. Instead of "timing of imaging", I suggest using the "phase of migraine cycle". Regarding the second part of the sentence, I am not sure whether the authors referred to specific phases (some studies assessing interictal stage, other studies the ictal stage...) or poorly defined phases, i.e., whether it was unclear that the patients were in interictal stage, or the sample included patients at different stages of the cycle. This should be clarified. Perhaps, I would also clarify that results from different phases of the cycle can lead to opposite trends (in other words, define what "inconsistency" means). Moreover, I would use the past to refer to direct results from the study (applicable to other parts of the Discussion).

28. Methodological considerations. Certainly, the authors made an excellent control of the migraine phase in this study, but this study performed no better control of other variables such as sex (not the first study that included only women in the sample), and the age was not really matched according to the distributions provided by the authors. Thus, I would highlight the control of the migraine phase or the hormonal state, but not the other variables, although it could be mentioned that only women and an age-balanced sample were employed.

29. Lines 321-322. The study by Jensen et al. (2005) assessed healthy subjects, no patients with episodic migraine. This must be corrected. Regarding references, it is not strictly necessary to cite a migraine paper to demonstrate advantages of DKI. In fact, the authors can highlight their study to remark that this study showed that DKI can detect changes unidentified with DTI.

Tables and Figures

30. Figure 1. I suggest adding the number of subjects for each group and subgroup. For the HC, it would be 15, and 15 for postov and pm. For M, it would be 14, and the numbers detailed in page 8. The discarded ictal session should be directly excluded or shown as excluded in the Figure. The information should be added in the caption accordingly (not the whole description, but just state that the number of subjects per group and session is shown).

31. Table 1. I think the "Comparisons" row has wrong values in all comparisons except M-inter vs HC-postov. In this case, there is a column for DTI and another column for DKI, but for the remaining comparisons, there are two columns either for DTI or DKI. This must be corrected or clarified. Furthermore, could the authors better adjust the first row to be approximately in the centre of the two columns used in the following rows? Currently, the title of each comparison, particularly from M-pre vs. HC-pm is a bit right-shifted. Separately, to how many voxels corresponded a 0.1% (DKI, reduced AD in M-ict compared to HC-pm)? This seems a very low number of voxels, but Figure 3 showed a distinguishable area with statistically significant differences.

32. Figures 5-6. Were the p-values (e.g., p < 0.05) obtained after adjustment or before adjustment for multiple comparisons? If they were already adjusted, just clarify in the caption that p-values were adjusted. Otherwise, the authors must use the corrected p-values and repeat the figures if necessary, adding in the caption the clarification of the adjusted p-values.

33. Figure S3. If possible, the authors should use the Greek letter "ρ" instead of R, as Spearman's correlation coefficient was employed. Moreover, could the authors use a figure with better resolution?

Use of English and others

34. I suggest using "changes" instead of "alteration" to refer to the white matter changes throughout the text (totally optional).

35. Line 83. Does the "(25)" mean something?

36. There are separate words written together in the text (Page 4, Materials and Methods). The authors must check the text to correct this issue (e.g., "headachesociety") and minor grammar mistakes.

37. Page 5, Lines 110-115. To list the diverse migraine phases, "1)" and "2)" were used, but for M-ict and M-post there were no numbers, so "3)" and "4)" must be added, or the style should be corrected according to the authors' preferences. In Line 113, the authors can write "which was confirmed" (as in Line 115) or just "confirmed", but not "which confirmed". The authors must replace "ICDH" by "ICHD-3".

38. Line 204. I think the authors wrote separate sentences, when there should be only one.

39. Line 221. "Parameters" instead of "parameter". Moreover, in the same subsection, "five ROIs" (and equivalent situations) should be used instead of "5 ROIs". In line 226, define what "cingulate" stands for.

40. Line 238. I miss a connector (e.g., "In this study,...") or a sentence to introduce the results ("The main finding of our study was...").

## References

[B1] Ades-Aron B, Ashina S, Conti B, Lui YW, Minen M, Novikov DS, Shepherd T, Fieremans E (2019) White matter microstructural alterations in chronic, episodic, and aura migraine In: proc. international society for magnetic resonance in medicine (ISMRM). Med. 27:293.

[B2] Ades-Aron B, Veraart J, Kochunov P, McGuire S, Sherman P, Kellner E, Novikov DS, Fieremans E (2018) Evaluation of the accuracy and precision of the diffusion parameter EStImation with Gibbs and NoisE removal pipeline. Neuroimage 183:532–543. 10.1016/j.neuroimage.2018.07.066 30077743 PMC6371781

[B3] Ambrosini A, Rossi P, De Pasqua V, Pierelli F, Schoenen J (2003) Lack of habituation causes high intensity dependence of auditory evoked cortical potentials in migraine. Brain 126:2009–2011. 10.1093/brain/awg206 12821515

[B4] Andersson JLR, Skare S, Ashburner J (2003) How to correct susceptibility distortions in spin-echo echo-planar images: application to diffusion tensor imaging. Neuroimage 20:870–888. 10.1016/S1053-8119(03)00336-7 14568458

[B5] Andersson JLR, Sotiropoulos SN (2015) Non-parametric representation and prediction of single- and multi-shell diffusion-weighted MRI data using Gaussian processes. Neuroimage 122:166–176. 10.1016/j.neuroimage.2015.07.067 26236030 PMC4627362

[B6] Benedetti F, et al. (2011) Disruption of white matter integrity in bipolar depression as a possible structural marker of illness. Biol Psychiatry 69:309–317. 10.1016/j.biopsych.2010.07.028 20926068

[B7] Chong CD, Schwedt TJ (2015) Migraine affects white-matter tract integrity: a diffusion-tensor imaging study. Cephalalgia 35:1162–1171. 10.1177/0333102415573513 25712998

[B8] Coppola G, et al. (2014) Dynamic changes in thalamic microstructure of migraine without aura patients: a diffusion tensor magnetic resonance imaging study. Eur J Neurol 21:287–292. 10.1111/ene.12296 24200371

[B9] DaSilva AFM (2007) Interictal alterations of the trigeminal somatosensory pathway and PAG in migraine. Neuroreport 18:301–305. 10.1097/WNR.0b013e32801776bb 17435592 PMC3745625

[B10] Davis SW, Dennis NA, Buchler NG, White LE, Madden DJ, Cabeza R (2009) Assessing the effects of age on long white matter tracts using diffusion tensor tractography. Neuroimage 46:530–541. 10.1016/j.neuroimage.2009.01.068 19385018 PMC2775533

[B11] Dubol M, Epperson CN, Sacher J, Pletzer B, Derntl B, Lanzenberger R, Sundström-Poromaa I, Comasco E (2021) Neuroimaging the menstrual cycle: a multimodal systematic review. Front Neuroendocrinol 60:100916. 10.1016/j.yfrne.2020.100878 33098847

[B12] Goadsby PJ, Holland PR, Martins-Oliveira M, Hoffmann J, Schankin C, Akerman S (2017) Pathophysiology of migraine: a disorder of sensory processing. Physiol Rev 97:553–622. 10.1152/physrev.00034.2015 28179394 PMC5539409

[B13] Gomez-Pilar J, Martínez-Cagigal V, García-Azorín D, Gómez C, Guerrero Á, Hornero R (2022) Headache-related circuits and high frequencies evaluated by EEG, MRI, PET as potential biomarkers to differentiate chronic and episodic migraine: evidence from a systematic review. J Headache Pain 23:95. 10.1186/s10194-022-01465-1 35927625 PMC9354370

[B14] Hua K, Zhang J, Wakana S, Jiang H, Li X, Reich DS, Calabresi PA, Pekar JJ, van Zijl PCM, Mori S (2008) Tract probability maps in stereotaxic spaces: analyses of white matter anatomy and tract-specific quantification. Neuroimage 39:336–347. 10.1016/j.neuroimage.2007.07.053 17931890 PMC2724595

[B15] Inano S, Takao H, Hayashi N, Abe O, Ohtomo K (2011) Effects of age and gender on white matter integrity. Am J Neuroradiol 32:2103–2109. 10.3174/ajnr.A2785 21998104 PMC7964377

[B16] Ito K, Kudo M, Sasaki M, Saito A, Yamashita F, Harada T, Yokosawa S, Uwano I, Kameda H, Terayama Y (2016) Detection of changes in the periaqueductal gray matter of patients with episodic migraine using quantitative diffusion kurtosis imaging: preliminary findings. Neuroradiology 58:115–120. 10.1007/s00234-015-1603-8 26446146

[B17] Jensen JH, Helpern JA, Ramani A, Lu H, Kaczynski K (2005) Diffusional kurtosis imaging: the quantification of non-Gaussian water diffusion by means of magnetic resonance imaging. Magn Reson Med 53:1432–1440. 10.1002/mrm.20508 15906300

[B18] Karsan N, Goadsby PJ (2023) Neuroimaging in the pre-ictal or premonitory phase of migraine: a narrative review. J Headache Pain 24:106. 10.1186/s10194-023-01617-x 37563570 PMC10416375

[B19] Kellner E, Dhital B, Kiselev VG, Reisert M (2016) Gibbs-ringing artifact removal based on local subvoxel-shifts. Magn Reson Med 76:1574–1581. 10.1002/mrm.26054 26745823

[B20] Koay CG, Basser PJ (2006) Analytically exact correction scheme for signal extraction from noisy magnitude MR signals. J Magn Reson 179:317–322. 10.1016/j.jmr.2006.01.016 16488635

[B21] Li XL, Fang YN, Gao QC, Lin EJ, Hu SH, Ren L, Ding MH, Luo BN (2011) A diffusion tensor magnetic resonance imaging study of corpus callosum from adult patients with migraine complicated with depressive/anxious disorder. Headache 51:237–245. 10.1111/j.1526-4610.2010.01774.x 20946428

[B22] Liu J, et al. (2013) Migraine-related gray matter and white matter changes at a 1-year follow-up evaluation. J Pain 14:1703–1708. 10.1016/j.jpain.2013.08.013 24290450

[B23] Marciszewski KK, Meylakh N, Di Pietro F, Macefield VG, Macey PM, Henderson LA (2019) Fluctuating regional brainstem diffusion imaging measures of microstructure across the migraine cycle. eNeuro 6:ENEURO.0005-19.2019. 10.1523/ENEURO.0005-19.2019 31300542 PMC6658917

[B24] May A (2009) New insights into headache: an update on functional and structural imaging findings. Nat Rev Neurol 5:199–209. 10.1038/nrneurol.2009.28 19347025

[B25] Messina R, Rocca MA, Colombo B, Pagani E, Falini A, Comi G, Filippi M (2015) White matter microstructure abnormalities in pediatric migraine patients. Cephalalgia 35:1278–1286. 10.1177/0333102415578428 25795038

[B26] Nichols TE, Holmes AP (2002) Nonparametric permutation tests for functional neuroimaging: a primer with examples. Hum Brain Mapp 15:1–25. 10.1002/hbm.1058 11747097 PMC6871862

[B27] Olesen J (2018) Headache classification committee of the International Headache Society (IHS) the international classification of headache disorders, 3rd edition. Cephalalgia 38:1–211. 10.1177/0333102417738202 29368949

[B28] Olson IR, Von Der Heide RJ, Alm KH, Vyas G (2015) Development of the uncinate fasciculus: implications for theory and developmental disorders. Dev Cogn Neurosci 14:50–61. 10.1016/j.dcn.2015.06.003 26143154 PMC4795006

[B29] Peng K-P, May A (2020) Redefining migraine phases - a suggestion based on clinical, physiological, and functional imaging evidence. Cephalalgia 40:866–870. 10.1177/0333102419898868 31928343 PMC7366426

[B30] Petrušić I, Daković M, Kačar K, Mićić O, Zidverc-Trajković J (2018) Migraine with aura and white matter tract changes. Acta Neurol Belg 118:485–491. 10.1007/s13760-018-0984-y 30006859

[B31] Pierpaoli C, Jezzard P, Basser PJ, Barnett A, Di Chiro G (1996) Diffusion tensor MR imaging of the human brain. Radiology 201:637–648. 10.1148/radiology.201.3.8939209 8939209

[B32] Rahimi R, Dolatshahi M, Abbasi-Feijani F, Momtazmanesh S, Cattarinussi G, Aarabi MH, Pini L (2022) Microstructural white matter alterations associated with migraine headaches: a systematic review of diffusion tensor imaging studies. Brain Imaging Behav 16:2375–2401. 10.1007/s11682-022-00690-1 35710680 PMC9581876

[B33] Rolls ET (2019) The cingulate cortex and limbic systems for emotion, action, and memory. Brain Struct Funct 224:3001–3018. 10.1007/s00429-019-01945-2 31451898 PMC6875144

[B34] Schramm S, Börner C, Reichert M, Baum T, Zimmer C, Heinen F, Bonfert MV, Sollmann N (2023) Functional magnetic resonance imaging in migraine: a systematic review. Cephalalgia 43:033310242211282. 10.1177/03331024221128278 36751858

[B35] Schulte LH, Mehnert J, May A (2020) Longitudinal neuroimaging over 30 days: temporal characteristics of migraine. Ann Neurol 87:646–651. 10.1002/ana.25697 32031707

[B36] Shibata Y, Ishiyama S, Matsushita A (2018) White matter diffusion abnormalities in migraine and medication overuse headache: a 1.5-T tract-based spatial statistics study. Clin Neurol Neurosurg 174:167–173. 10.1016/j.clineuro.2018.09.022 30245435

[B37] Shua N, Wang Z, Qib Z, Li K, Hea Y (2011) Multiple diffusion indices reveals white matter degeneration in Alzheimer’s disease and mild cognitive impairment: a tract-based spatial statistics study. Adv Alzheimer Dis 26:275–285. 10.3233/JAD-2011-0024 21971467

[B38] Smith SM, et al. (2006) Tract-based spatial statistics: voxelwise analysis of multi-subject diffusion data. Neuroimage 31:1487–1505. 10.1016/j.neuroimage.2006.02.024 16624579

[B39] Smith SM, Nichols TE (2009) Threshold-free cluster enhancement: addressing problems of smoothing, threshold dependence and localisation in cluster inference. Neuroimage 44:83–98. 10.1016/j.neuroimage.2008.03.061 18501637

[B40] Spielberger CD (1970) Manual for the state-trait anxiety inventory (self-evaluation questionnaire).

[B41] Stankewitz A, Aderjan D, Eippert F, May A (2011) Trigeminal nociceptive transmission in migraineurs predicts migraine attacks. J Neurosci 31:1937–1943. 10.1523/JNEUROSCI.4496-10.2011 21307231 PMC6633029

[B42] Stankewitz A, Schulz E (2022) Intrinsic network connectivity reflects the cyclic trajectory of migraine attacks. Neurobiol Pain 11:100985. 10.1016/j.ynpai.2022.100085 35243179 PMC8861450

[B43] Szabó N, et al. (2018) Evidence for plastic processes in migraine with aura: a diffusion weighted MRI study. Front Neuroanat 11:138. 10.3389/fnana.2017.00138 29387002 PMC5776127

[B44] Tournier JD, Smith R, Raffelt D, Tabbara R, Dhollander T, Pietsch M, Christiaens D, Jeurissen B, Yeh CH, Connelly A (2019) MRtrix3: a fast, flexible and open software framework for medical image processing and visualisation. Neuroimage 202:116137. 10.1016/j.neuroimage.2019.116137 31473352

[B45] Veraart J, Novikov DS, Christiaens D, Ades-aron B, Sijbers J, Fieremans E (2016) Denoising of diffusion MRI using random matrix theory. Neuroimage 142:394–406. 10.1016/j.neuroimage.2016.08.016 27523449 PMC5159209

[B46] Von Der Heide RJ, Skipper LM, Klobusicky E, Olson IR (2013) Dissecting the uncinate fasciculus: disorders, controversies and a hypothesis. Brain 136:1692–1707. 10.1093/brain/awt094 23649697 PMC3673595

[B47] Winkler AM, Ridgway GR, Webster MA, Smith SM, Nichols TE (2014) Permutation inference for the general linear model. Neuroimage 92:381–397. 10.1016/j.neuroimage.2014.01.060 24530839 PMC4010955

[B48] Winston GP (2012) The physical and biological basis of quantitative parameters derived from diffusion MRI. Quant Imaging Med Surg 2:254. 10.3978/j.issn.2223-4292.2012.12.05 23289085 PMC3533595

[B49] Yu D, et al. (2013a) Axonal loss of white matter in migraine without aura: a tract-based spatial statistics study. Cephalalgia 33:34–42. 10.1177/0333102412466964 23150889

[B50] Yu D, et al. (2013b) White matter integrity affected by depressive symptoms in migraine without aura: a tract-based spatial statistics study. NMR Biomed 26:1103–1112. 10.1002/nbm.2924 23447382

[B51] Zung WWK, Richards CB, Short MJ (1965) Self-rating depression scale in an outpatient clinic: further validation of the SDS. Arch Gen Psychiatry 13:508–515. 10.1001/archpsyc.1965.01730060026004 4378854

